# Promising Technological and Industrial Applications of Deep Eutectic Systems

**DOI:** 10.3390/ma14102494

**Published:** 2021-05-12

**Authors:** Alberto Mannu, Marco Blangetti, Salvatore Baldino, Cristina Prandi

**Affiliations:** Department of Chemistry, University of Turin, Via Pietro Giuria 7, I-10125 Turin, Italy; marco.blangetti@unito.it (M.B.); salvatore.baldino@unito.it (S.B.)

**Keywords:** deep eutectic solvents, materials, API formulation, gas sorbents, ionothermal synthesis, organic synthesis

## Abstract

Deep Eutectic Systems (DESs) are obtained by combining Hydrogen Bond Acceptors (HBAs) and Hydrogen Bond Donors (HBDs) in specific molar ratios. Since their first appearance in the literature in 2003, they have shown a wide range of applications, ranging from the selective extraction of biomass or metals to medicine, as well as from pollution control systems to catalytic active solvents and co-solvents. The very peculiar physical properties of DESs, such as the elevated density and viscosity, reduced conductivity, improved solvent ability and a peculiar optical behavior, can be exploited for engineering modular systems which cannot be obtained with other non-eutectic mixtures. In the present review, selected DESs research fields, as their use in materials synthesis, as solvents for volatile organic compounds, as ingredients in pharmaceutical formulations and as active solvents and cosolvents in organic synthesis, are reported and discussed in terms of application and future perspectives.

## 1. Introduction

The so-called Deep Eutectic Solvents (or DESs) have been firstly described in the scientific literature in 2003 with the pioneering work of Abbot and co-workers on the eutectic behavior of urea in the presence of some quaternary ammonium salts [1].

Since this seminal report, many efforts have been devoted in the last decade to the development of such systems which revealed impressive performances when used as green solvents. In particular, their peculiar physical properties, such as low volatility, flammability and vapor pressure, increased chemical and thermal stability could be exploited for generating a superior class of improved solvents [2]. In addition, by designing DESs with specific components, it is possible to obtain eutectic mixtures with low toxicity and high biodegradability [3].

Generally speaking, a DES is obtained by combining in eutectic molar ratio specific Hydrogen bond Donors (HBDs) and Acceptors (HBAs). The most evident, as easily visible, parameter related with the formation of a DES is represented by its melting point, which results lower than the one of the formers HBDs and HBAs, typically with values around the room temperature. As an example, when the quaternary ammonium salt choline chloride is mixed with urea (both solid at room temperature) in a 1:2 molar ratio, a viscous liquid is obtained in a few minutes. When all the possible combinations between any HBA and HBD are considered, the definition of DES becomes tricky, especially in terms of distinction between Deep Eutectic (DES) and Eutectic Solvents (ES). This matter has been the subject of research and discussion for many years. Currently, according to Ryder [4] and Martins [5], the term *deep* should be associated with eutectic mixtures that show a decreasing of the melting point with respect to the ideal eutectic point and not with respect to the melting points of the former HBA and HBD. In the absence of such a condition, a simple eutectic mixture (ES) is thus obtained.

Regarding the possibility to classify a DES according to the nature of the formers HBA and HBD, Abbott and co-workers [6] defined Deep Eutectic Solvents using the general formula:

Cat^+^X^−^ · Y^−^ (1)

where Cat^+^ is an organic cation (typically an ammonium, but also phosphonium or sulphonium are included), X^−^ is the Lewis base counterion (generally a halide ion) and Y represents a Lewis or Brønsted acid which is involved in the formation of the anionic complex with X^−^. Nowadays, the majority of DESs that have been prepared and studied could be classified into five different classes, as shown in Table 1.

Type I and Type II DESs combine a quaternary ammonium salt and a metal chloride, the latter both in anhydrous (Type I) or in hydrated form (Type II). DESs can be formulated by the combination of a quaternary ammonium salt with a HBD, as in the above mentioned ChCl-based DES, where the HBD species is typically a small organic molecule (Type III DESs). Type IV, a combination between Type II and Type III DESs, includes the deep eutectic mixtures formed by a metal chloride hydrate and an organic HBD [6]. Recently, a fifth class of DES has been integrated in the general classification including all the deep eutectics composed of only non-ionic, molecular HBAs and HBDs (Type V DESs) [7].

**Figure 1:**
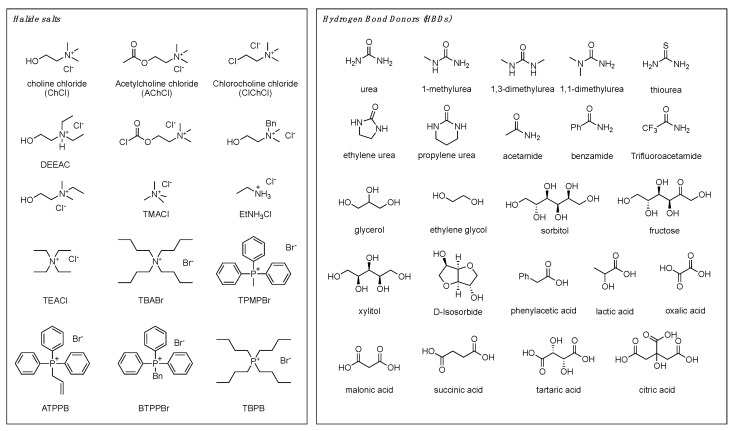
Selected examples of HBAs and HBDs for the formulation of DESs. Bn = benzyl, Ph = phenyl.

Type III DESs are the most investigated class of DESs. The HBA is typically an ionic halide salt, such as an ammonium or phosphonium salt. Choline chloride (ChCl), a quaternary ammonium salt, is one of the most employed HBAs for the formation of DESs, since it fulfils several sustainability principles due to its reduced costs, high biodegradability, low toxicity and bioavailability. The other component of the DES is a generally safe and bioavailable small molecule as urea, organic carboxylic acids (e.g., mono- or bicarboxylic acids, citric acid or aminoacids) or polyols (e.g., glycerol, ethylene glycol or carbohydrates). Some selected examples of HBAs and HBDs are illustrated in Figure 1.

As a matter of fact, the almost unlimited possible combinations and the large pool of bio-derived and bio-inspired components available offer boundless possibilities to formulate new DESs with remarkable properties in terms of physico-chemical parameters. A relevant complementary class of DESs is represented by Natural Deep Eutectic Solvents (NADESs). The term NADES is generally intended to designate DESs composed only by naturally occurring compounds. In 2011 Verpoorte observed that a small number of primary metabolites as carboxylic acids, choline, sugars and aminoacids are present in high amounts in living organisms, much more abundant than expected on the basis of their metabolic roles [8]. It has also been proposed that NADESs may be involved in the resistance of some organisms to low temperatures and drought [9,10]. The hypothesis put forward to explain the ubiquitous presence of NADESs in living organisms is that the mixture of metabolites could form eutectic mixtures that would serve as reaction media for the biosynthesis of non-water-soluble molecules [8].

Taking inspiration from nature, several eutectic mixtures of bioderived compounds have been prepared and employed for various applications [11,12,13]. Among the wide variety in terms of components of NADESs, hydrophobic mixtures based on terpenes and fatty acids have been reported [14,15,16,17]. A growing number of publications report on very diverse use of NADESs. The special features of NADESs, such as biodegradability and biocompatibility [11], suggest that they are alternative candidates for concepts and applications and can be used in replacement to Volatile Organic Solvents (VOCs) in organic synthesis as well as in extraction processes [2,18,19]. Stemming from the idea that NADESs are the natural environment for metabolic processes, biocatalysis in NADESs has received much attention for those transformations in which substrates or products show low water solubility [20,21,22]. Most of the applications rely on the use of NADESs as extraction media for Natural Products (NPs). As NADES species exhibit a superior solubilizing ability for NPs, this provides a special advantage for NADESs as extraction media. Examples in the extraction capabilities of NADESs for a large number of natural products are covered in a pair of excellent recent reviews [23], and more recent references [24,25,26,27,28].

Further exploitation of NADES properties refers to their use as drug carriers. A recent study refers to the slightly reduced permeability of chloramphenicol through the pig skin after NADES application, which most likely was caused by hydrogen bonding between the NADES and proteins in the skin based on FT-IR-results [29]. Additional areas of possible further applications of NADES including their use as reaction, extraction and chromatographic media as well as their biomedical relevance are expected in the next future as far as the complexity of their supramolecular properties is elucidated.

The intriguing properties of DES make them exploitable not only as green solvents, but also in other fields of industrial interest. In this context, cutting edge applications of DESs are emerging, from the designing of functional materials [30], to the development of innovative medicinal formulations, to the possibility to act as active catalytic systems [31]. A relevant research line with important industrial applications is related with the employment of DESs as green solvents for biomass processing. This specific topic has been recently reviewed in a very exhaustive way by Jablonský and coworkers and thus will not be discussed herein [32].

In the present review, an overview about the application of DESs as possible organic liquid semiconductors, as shape directing agents, as solvents for VOCs and as additive or solvents in pharmaceutical formulations, is reported. In particular, a special focus on the molecular structure of these systems and the consequences on their performances will be given.

The present review has been divided into five parts.

In the first section, the relationship between the physical properties of DESs and their molecular structure will be discussed in general terms. Common preparation procedures will be also considered, as well as the low stability of some common DESs, recently highlighted by some Authors. In the second section, the application of eutectics in ionothermal synthesis and thus to the production of materials will be described. In the third part, the employment of DESs-based devices to the solubilization of Volatile Organic Compounds (VOCs) will be discussed. In the fourth section, promising application of some DESs in medicine (Therapeutic DESs, THEDESs), as in the formulation of improved drugs will be presented. In the last part, the possibility to employ DESs as non-innocent solvents in organic synthesis will be reported.

## 2. Molecular Structure of Deep Eutectic Systems and Properties

The so-called deep eutectic solvents (DESs) were initially considered as sort of room temperature ionic liquids [33]. Despite this first raw classification, DESs show many differences from the parent ionic liquids. DESs are not liquid salts, being the components very often neutral small molecules. They are in fact formed by eutectic combinations of HBAs and HBDs connected through an intense hydrogen bond network responsible for a very peculiar supramolecular architecture. In addition, the DESs’ physical properties are uncommon as their enhanced solvent ability. Indeed, the possibility to produce sustainable DESs (as NADESs, by choosing appropriate HBAs and HBDs), make them potentially high performant green solvents. Nevertheless, during the last 15 years, an increasing number of alternative applications has been reported for many DESs, and some Authors started to use the term “DES” as short name for deep eutectic systems, in this way comprising any possible exploitation of such compounds. By the way, these eutectic systems have grown in importance as they show some peculiar physical properties directly related to their molecular structure. Several studies have been reported in order to characterize and classify many DESs considering some physical parameter, such as density, viscosity or conductivity. In addition, many experimental and theoretical attempts have been done in order to explain the DESs behavior at molecular level. A DES is usually characterized by a decreased viscosity, an increased density, a low conductivity and it usually shows liquid consistency at room temperature. In particular, when its melting point is plotted versus the molar fraction between the former constituents, a drop is experimentally observed in correspondence of the eutectic composition. When the drop is deeper than the expected theoretical melting point, the term DES results appropriately. Otherwise, the resulting mixture should be addressed as a simple ES (eutectic system) [4,34]. Many researchers studied the variation of physical properties as density and viscosity [35,36,37,38], refractive index [39] and speed of sound [40] of a DES and how these can be tailored by changing the nature of HBA, HBD or their composition. The possibility to engineer such systems makes of high priority the understanding of the supramolecular interactions which govern the formation of the DES.

In order to explain how the molecular structure and interactions are related to the experimentally observed melting point depression and to the specific physical characteristics commonly described, Abbot proposed the extension of the hole theory of Fürth to such systems [41]. In general, hole models are based on the statement that the vacancies present at molecular level are randomly distributed [42]. When an ionic mixture melts, the variation in temperature during the melting process produces fluctuations of the local density, which are responsible for the formation of empty spaces [43]. The consequent molecular framework is dynamic and allows, at some specific conditions, the constant movement of the ions with opportune size to fit in the holes, which move by this mechanism all-over the network. By exploiting the equations described by Abbot and co-workers, it is possible to determine the average size of the holes and to relate it with density and viscosity [9]. In particular, the relationship between the volume of the vacancies and the superficial tension, can be exploited to predict which HBAs and HBDs are more appropriated for customizing DESs with specific properties [44].

**Figure 2:**
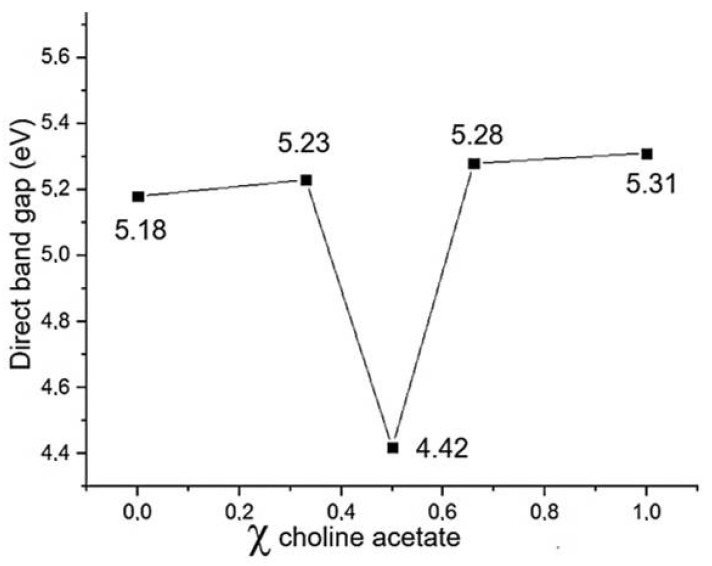
Variation of the band gap energy as function of the molar ratio between choline acetate and levulinic acid. Figure made from the data reported in reference [45].

Recently, some attempts to compare the molecular structure of different DESs by meaning of their UV-VIS profile have been reported. Through the UV-VIS based Tauc plot method, the Band Gap (BG) energy of several hydrophilic choline-based [45] or hydrophobic phosphonium-based DESs [46] were determined and compared, revealing a relationship between the BG and the eutectic composition, which is analogue to the one observed when the melting point is expressed in terms of molar ratio between HBA and HBD (Table 2 and Figure 2).

The plot reported in Figure 2 represents the variation of the band gap energy as function of the molar fraction of choline acetate for the system choline acetate/levulinic acid. When the system contains equal moles of both the constituents, a drop of the bang gap energy is experimentally observed. It has been reported for several systems that the composition at which the band gap energy deeply decreases corresponds to the eutectic ratio between the formers HBAs and HBDs.

This UV-VIS-based procedure represents a fast methodology, alternative to the determination of the melting point, in order to find the correct eutectic composition of HBAs and HBDs mixtures. In addition, a relevant effect of the water on the BG energy was observed: the addition of 10% of water to choline based DESs resulted in a reduction of the BG energy. It is known that water addition on DESs reduces the lattice energy and this in part explains the trend observed. The possibility to tune some features of eutectic mixtures by adding specific amounts of water has been an object of intensive research activity, and many experimental and theoretical studies have been focused on the structural effect of water addition on eutectics [47]. It has been demonstrated that even low amounts of water affect sensibly the properties of a DES, but in order to produce a relevant structural variation, highly amount of water should be added. Hammond and co-workers [48] studied the water tolerance of the eutectic system composed by choline chloride and urea (1:2), revealing that only after a water addition superior to 42 wt% the system shows a transition from an ionic mixture to an aqueous solution, ceasing to exist in form of hydrated DES. At molecular level, it has been observed that the water tolerance is related to the DES nanostructure, which can host water molecules up to certain amounts without producing relevant structural changes [49].

Working below the limit of tolerance, it is possible to take advantage by the addition of water to a DES. One example of this can be represented by the choline chloride/urea (1:2) DES, which revealed to change its capacity to solubilize the CO_2_ by adding even small amounts of water [50].

Even though the know-how about the specific effect of water content on the interaction between HBAs and HBDs is important for designing DESs with improved performances, the intrinsic hygroscopicity of eutectics is even more important. In fact, when DESs are used in industrial processes, the uptake of water from air cannot be avoided and, in this context, water is usually considered as an impurity [51]. Chen and co-workers determined the amount of water adsorbed (after 8 h) by a series of choline chloride based DESs containing as HBD glutaric acid, glycerol, ethylene glycol, xylitol, urea, glucose, methyl urea and oxalic acid. Most hygroscopic DESs (containing glycerol or ethylene glycol) showed not negligible adsorption values, in the range of 5–7 wt% [52]. As discussed before, these values are not high enough to disrupt the deep eutectic nature of the DES, nevertheless they must be taken into account in terms of influence on the DES physical properties. An exhaustive review paper concerning the effect of the water on specific DESs has been published in 2019 by El Achkar and co-workers [53].

As a matter of fact, the addition of water to a binary DES can be considered as the conversion of the original binary system in a new ternary one, where the water acts as a second HBD/HBA. This aspect is of general interest as the understanding of the combined effect of one HBAs and two HBDs on the properties of the resulting ionic mixture is pivotal for achieving a customization capacity. Recently, the effects of the preparation procedure, temperature and addition of a second HBD (water, methanol, 2-propanol, glycerol) were assessed for ternary mixtures containing choline chloride and ethylene glycol [54]. The Authors described an important effect of the time in the assessment of the DES structure after its preparation, indicating a fast ageing of these mixtures which should be considered for their application.

A further aspect of interest is related to the solvation of the anion in choline chloride based DESs. In fact, by ^35^Cl NMR spectroscopy it is possible to describe the behavior of the anion in the presence of different amounts of water. Gabriele and coworkers correlated the ^35^Cl linewidth with the increasing of weakness of DES-DES H-bond on samples of choline chloride/glycol (glycol = diethylene glycol, triethylene glycol, polyethylene glycol 200) upon addition of increasing amounts of water [55]. The same technology was employed by Di Pietro et al., who conducted ^35^Cl NMR measurements on choline chloride based DESs (choline chloride/urea or glycolic acid) combined with theoretical studies and described the variation of the chloride solvation with the hydration of the system [56].

Each aspect related to the molecular structure of DESs and its consequences on their physical properties and thus on their possible applications should be contextualized in terms of operational window. In fact, DESs are not free from degradation, due to the simple fact that their components may decompose and/or react with each other. This possible issue, being reliable the purity of the single components, may basically occur in two moments: during and after the preparation of the eutectic mixture, depending, respectively, on the mixing and storage conditions employed. It has been reported that DESs usually display a thermal stability which is intermediate between their pure components [57,58,59], and HBDs are in general less thermally stable than the HBA. To the best of our knowledge, the possibility of a thermal degradation of DESs has been considered only for the systems ChCl/polyols and ChCl/carboxylic acids [60,61,62], whereas there are no reports on the thermal behavior of metal-based DESs.

**Scheme 1:**
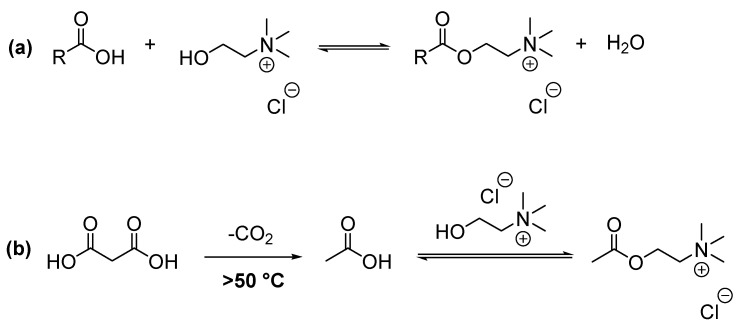
Esterification reaction between the ChCl and the acid component in the DES (**a**) and parallel degradation pathway of MA in ChCl/MA (**b**).

The ChCl/carboxylic acid (e.g., oxalic, malic, malonic, levulinic acid) DESs appear to be the most sensitive systems compared with ChCl/polyols. As a matter of fact, all the evidence present in the literature has shown decomposition of the HBDs within the eutectic mixture even at relatively low temperatures (50–60 °C) [60,61]. The decomposition pathway observed consisted in the esterification between the HBD and ChCl, as inferred from ^1^H NMR measurements, likely promoted by the acidic environment of the DES. Heating the mixtures at different temperatures (60–100 °C) for 2 h (Table 3) involves an increase of both the amount of ester product and water in the mixture (Scheme 1a), as measured by Karl-Fischer titration [61]. Apparently, the degradation process takes place regardless the preparation procedure employed, only at room temperature it occurs at much lower rate [60,61].

In addition, DESs with a bicarboxylic acid component such as ChCl/malonic acid (MA) easily undergoes decarboxylation of MA to acetic acid, even occurring during its preparation if the operating temperature is above 50 °C, as demonstrated independently by Gontrani et al. [60] and Rodriguez Rodriguez et al. [61] This additional degradation pathway may increase the deterioration of the DES through a further esterification reaction between ChCl and acetic acid (Scheme 1b).

**Scheme 2:**
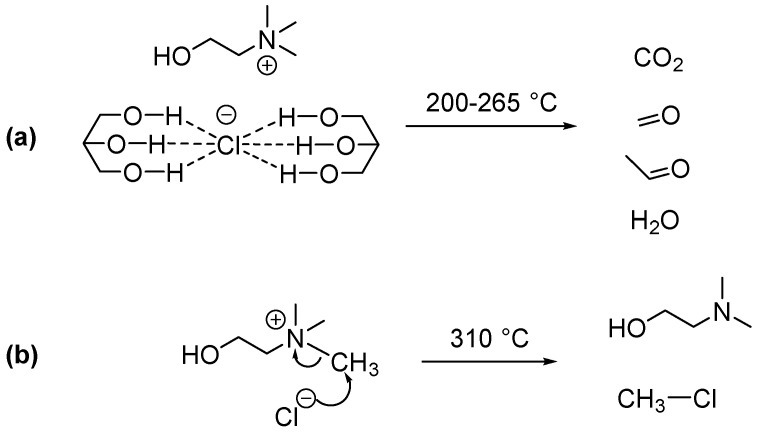
Degradation of ChCl/Gly (1:2) under TGA conditions: decomposition products of Gly (**a**) and ChCl (**b**) (assessed by FTIR measurements).

TGA studies demonstrated that degradation of ChCl/Gly (1:2) required elevated temperatures to occur [62]. FTIR data of the evolved gases suggest that over 200 °C CO_2_, formaldehyde, acetaldehyde and water are formed as degradation products of Gly, possibly due to an intramolecular redox reaction under the harsh conditions (Scheme 2a). On the other hand, the ChCl constituent decomposed at higher temperatures (>300 °C), in line with the usual greater resistance of the HBA. ChCl likely degrades to chloromethane obtained by nucleophilic attack of the chloride anion on the methyl group of the ammonium moiety, liberating *N*,*N*-dimethylaminoethanol (Scheme 2b).

## 3. DES for Ionothermal Synthesis

The know-how about the molecular structure of DESs can be exploited even for applications of industrial interest, as the synthesis of porous materials. Concerning this aspect, two subsequent key aspects should be considered: (I) the possibility to take advantage from the peculiar molecular structure of DES, (II) the chance to customize the DES structure by varying the nature of the HBA and HBD, and by modulating the preparation protocol.

In 2004 Cooper and co-workers reported an alternative protocol to the known hydrothermal synthesis for fabricating new zeotypes frameworks which was based on the unique properties on the DES formed by choline chloride and urea [63]. The Authors reported an innovative protocol for generate novel porous materials by exploiting the increased solvent ability of a DES combined with its well-established molecular structure, which resulted in being able to direct the synthesis toward specific spatial parameters. The possibility of using a DES not only as a solvent but also as Structure-Directing Agent (SDA) opened the way to several applied research lines. To date, many porous materials have been obtained in laboratory scale by exploiting specific combinations of HBA and HBD, and many review papers have well discussed the topic [64]. The process, named as ionothermal synthesis, can be performed with ionic liquids (ILs) or DESs and extended to the production of several porous materials (Table 4).

## 4. Deep Eutectic Solvents for Gas Solubilization

The exclusive structure of DESs, which can be enriched with many functional groups by choosing opportune combinations of HBAs and HBDs, can be exploited to increase their gas sorption ability. This topic is of particular interest as Volatile Organic Compounds (VOCs) are common by-products in many industrial processes [79] and they are considered as hazard chemicals often associated with many diseases [80]. VOCs are produced in large amounts in the transport sector, and they are common ingredients of many cleaning products, representing one of the major sources of air pollutants [81,82]. Reduction of VOCs emission represents a priority, and it has been considered mandatory by several national and international normative [83].

From a technological point of view, trapping VOCs from gas streams with liquid sorbents represents a very efficient way for decreasing their presence in the environment.

The effectiveness of such an approach is related to two main aspects: (I) the availability of high performant sorbents, (II) the sustainability of the developed sorbents. Since a few years ago, the approaches employed to solve the above-mentioned problems have been revealed to be not efficient. The employment of organic solvents for VOCs removal cannot be pursued due to the toxicity and pollution associated with the sorbent devices [84,85]. On the other hand, the development of water and water-based sorbent devices would represent the best green alternative. Unfortunately, this possibility is precluded by the low solubility of the hydrophobic moieties contained in VOCs in the presence of water. More recently, the possibility to exploit ionic liquids for trapping VOCs has been explored with good results [86]. Nevertheless, high prices associated with the complex synthetic conditions of ionic liquids drastically reduce the overall sustainability of the process [87]. In this context, a possible advancement of the current available technology for VOCs treatment can be represented by the use of DESs. As discussed above, DESs are generally cheap, low pollutant, liquid at room temperature and they strongly interact with organic molecules. Theoretically, the design of highly efficient DESs- based VOCs sorbents constitutes the best technological solution to air pollution. Early studies about the employment of ammonium-based eutectics as solvent for the CO_2_ were reported since 2015 (Li [88], Mirza [89], Leron [90], Lu [91]) and the technology was extended to other VOCs two years later by Moura and co-workers [92].

In particular, Moura reported the possibility to use both hydrophilic choline chloride-based and hydrophobic tetrabutylphosphonium bromide-based DESs as adsorbents for toluene, acetaldehyde and dichloromethane (Table 5). Good results were reported, especially in the case of acetaldehyde which was adsorbed in percentages reaching the 99% with respect to the initial amount. Since these pioneering works, few extensions of the topic have been reported so far, especially in terms of VOC adsorbed.

Applications in SO_2_ adsorption have been reported with quaternary ammonium salts-based DESs (HBD = glycerol [93], levulinic acid [94], guaiacol or cardanol [95]), with some thiocyanate-based DESs [96], and by employing eutectics formed by betaine or L-carnitine and ethylene glycol [97].

Regarding the application of DESs to the sorption of ammonia, a consistent family of DESs has been developed in the last years. Akhmetshina et al. reported a study on the gas sorbent properties of the DES 1-butyl-3-methyl imidazolium methanesulfonate/urea toward ammonia, hydrogen sulfide and carbon anhydride, with good results relative to the sorption capacity of the ammonia [98]. This study follows the results presented by Zhong [99,100] about phenol-based ternary DESs, by Yang using the hybrid DES choline chloride/resorcinol/glycerol (1:3:5) [101], Deng with protic NH_4_SCN-based DESs [102] and Vorotyntsev who employed methanesulfonate-based DESs.

A different approach to the development of suitable DESs-based systems for trapping VOCs was reported by Di Pietro and co-workers [103]. The Authors demonstrated the possibility to change the ability of cyclodextrin to encapsulate toluene and aniline by performing the process in DES (choline chloride/urea 1:2). The reporting of this hybrid DES-cyclodextrin system opens to new possibilities of engineering by designing opportune eutectics and combining them with specific macromolecules.

Table 5 reports a resume of the main DESs employed as VOCs solvents and the corresponding adsorption capacity.

**Table 5:** Selected DESs employed as VOCs absorbents. The best adsorbing system in terms of temperature and pressure is herein reported.

VOC	DES ^a^	Adsorption Capacity	Reference
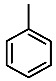	ChCl/U (1:2)	2.8 wt% ^b^ (303 K)	[92]
	ChCl/EG (1:2)	2.5 wt% ^b^ (333 K)	[92]
	ChCl/GLY (1:2)	2.1 wt% ^b^ (303 K)	[92]
	ChCl/LA (1:2)	2.9 wt% ^b^ (303 K)	[92]
	TBPB/GLY (1:1)	2.9 wt% ^b^ (303 K)	[92]
	TBPB/LA (1:6)	3.0 wt% ^b^ (303 K)	[92]
	TBPB/DA (1:2)	3.0 wt% ^b^ (303 K)	[92]
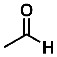	ChCl/U (1:2)	2.9 wt% ^b^ (303 K)	[92]
	ChCl/EG (1:2)	2.9 wt% ^b^ (303 K)	[92]
	ChCl/GLY (1:2)	2.9 wt% ^b^ (303 K)	[92]
	ChCl/LA (1:2)	2.9 wt% ^b^ (303 K)	[92]
	TBPB/GLY (1:1)	2.9 wt% ^b^ (303 K)	[92]
	TBPB/LA (1:6)	3.0 wt% ^b^ (303 K)	[92]
	TBPB/DA (1:2)	3.0 wt% ^b^ (303 K)	[92]
**CH_2_Cl_2_**	ChCl /U (1:2)	2.0 wt% ^b^ (303 K)	[92]
	ChCl/EG (1:2)	2.6 wt% ^b^ (333 K)	[92]
	ChCl/GLY (1:2)	2.1 wt% ^b^ (303 K)	[92]
	ChCl /LA (1:2)	2.8 wt% ^b^ (303 K)	[92]
	TBPB/GLY (1:1)	2.8 wt% ^b^ (333 K)	[92]
	TBPB/LA (1:6)	2.8 wt% ^b^ (303 K)	[92]
	TBPB/DA (1:2)	3.0 wt% ^b^ (303 K)	[92]
**CO_2_**	TBAB/TEA (1:5)	2.5 wt% ^b^ (303 K)	[88]
	TEAB/TEA (1:5)	2.9 wt% ^b^ (303 K)	[88]
	ChCl/MDEA (1:4)	3.0 wt% ^b^ (303 K)	[88]
	Ch/Cl/TEA (1:5)	3.0 wt% ^b^ (303 K)	[88]
	ChCl/MDEA (1:5)	4.0 wt% ^b^ (303 K)	[88]
	ChCl/TEA (1:4)	6.0 wt% ^b^ (303 K)	[88]
	ChCl/DEA (1:4)	15 wt% ^b^ (303 K)	[88]
	TBAB/MEA (1:5)	16 wt% ^b^ (303 K)	[88]
	TBAC/MEA (1:5)	17.5 wt% ^b^ (303 K)	[88]
	TEAB/MEA (1:5)	18 wt% ^b^ (303 K)	[88]
	TEAC/MEA (1:5)	22.5 wt% ^b^ (303 K)	[88]
	ChCl/MEA (1:4)	23 wt% ^b^ (303 K)	[88]
	TMAC/MEA (1:5)	23 wt% ^b^ (303 K)	[88]
	ChCl/MEA (1:5)	25 wt% ^b^ (303 K)	[88]
	TEAC/MEA/TEA	23 wt% ^b^ (303 K)	[88]
	ChCl/MEA/TEA	23 wt% ^b^ (303 K)	[88]
	TEAC/MEA/MDEA	23 wt% ^b^ (303 K)	[88]
	ChCl/MEA/MDEA	23 wt% ^b^ (303 K)	[88]
	TMAC/MEA/TEA	23 wt% ^b^ (303 K)	[88]
	TMAC/MEA/MDEA	30 wt% ^b^ (303 K)	[88]
	TMAC/MEA/FeCl_3_ (1:5:0.1)	25 wt% ^b^ (303 K)	[88]
	TMAC/MEA/CuCl_2_ (1:5:0.1)	26 wt% ^b^ (303 K)	[88]
	TMAC/MEA/NiCl_2_ (1:5:0.1)	26 wt% ^b^ (303 K)	[88]
	TMAC/MEA/CoCl_2_ (1:5:0.1)	26 wt% ^b^ (303 K)	[88]
	TMAC/MEA/NH_4_Cl (1:5:0.1)	28 wt% ^b^ (303 K)	[88]
	TMAC/MEA/ZnCl_2_ (1:5:0.1)	30 wt% ^b^ (303 K)	[88]
	TMAC/MEA/LiCl (1:5:0.1)	30 wt% ^b^ (303 K)	[88]
	ChCl/U (1:2)	3.559 mol_VOC_/kg_DES_ (303 K, 5.654 bar)	[90]
	1-butyl-3-methyl imidazolium methanesulfonate /U (1:1)	0.422 mol_VOC_/kg_DES_ (303 K, 6.984 bar)	[98]
**NEt_3_**	ChCl/PhOH/EG (1:5:4)	9.619 mol_VOC_/kg_DES_ (298 K, 101 kPa)	[99]
	ChCl/PhOH/EG (1:7:4)	7.652 mol_VOC_/kg_DES_ (313 K, 101 kPa)	[99]
	ChCl/U (1:2)	2.213 mol_VOC_/kg_DES_ (298 K, 95 kPa)	[100]
	ChCl/resorcinol/GLY (1:3:5)	9.982 mol_VOC_/kg_DES_ (298 K, 101 kPa)	[101]
	ChCl/*D*-fructose/GLY (1:3:5)	6.471 mol_VOC_/kg_DES_ (313 K, 101 kPa)	[101]
	NH_4_SCN/GLY (2:3)	10.353 mol_VOC_/kg_DES_ (298 K, 101 kPa)	[102]
**SO_2_**	ChCl/guaiacol (1:3; 1:4; 1:5)	0.528; 0.501; 0.479 gVOC for gDES	[95]
	ChCl/cardanol (1:3; 1:4; 1:5)	0.196; 0.170; 0.149 gVOC for gDES	[95]
	ChCl/LA (1:3)	0.557 gVOC for gDES	[94]
	TBAC/LA (1:3)	0.622 gVOC for gDES	[94]
	ChCl/EG (1:2)	2.25 mol SO_2_/mol DES	[104]
	ChCl/MA (1:1)	1.40 mol of SO_2_ mol DES	[104]
	ChCl/U (1:2)	1.57 mol of SO_2_ mol DES	[104]
	ChCl/ thiourea (1:1)	2.37 mol of SO_2_ mol DES	[104]
**NH_3_**	1-butyl-3-methyl imidazolium methanesulfonate /U (1:1)	4.150 mol_VOC_/kg_DES_ (313 K, 5.258 bar)	[98]
**H_2_S**	1-butyl-3-methyl imidazolium methanesulfonate /U (1:1)	1.034 mol_VOC_/kg_DES_ (303 K, 6.450 bar)	[98]

^a^ ChCl: choline chloride, U: urea, GLY: glycerol, EG: ethylene glycol, LA: lactic acid, DA: decanoic acid, TBPB: tetrabutylphosphonium bromide, TBAB: tetrabutylammonium bromide, TEA: triethylamine, TEAB: tetraethylammonium bromide, MDEA: methyldiethanolamine, MEA: methylethanolamine, TEAC: tetraethylammonium chloride, TMAC: tetramethylammonium chloride. ^b^ Data extrapolated from the plots reported by the Authors. The numbers reported in the table are approximated.

## 5. Deep Eutectic Solvents in Medicine

One of the major challenges of the pharmaceutical industry is the improvement of existing active pharmaceutical ingredients (APIs) in terms of efficiency and pharmacological action, which are strongly correlated with a multitude of physico-chemical parameters such as solubility, permeation and bioavailability [105,106]. This approach is crucial in view of the development of new therapeutic agents, since it suppresses the clinical trial costs required in the drug development process.

**Figure 3:**
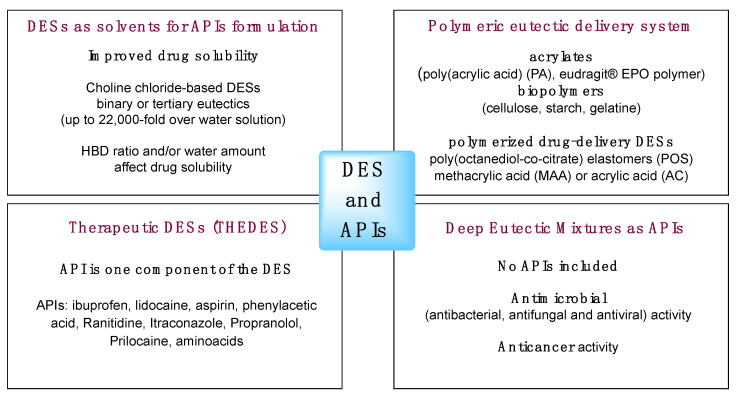
Pharmaceutical applications of Deep Eutectic Solvents: state-of-the-art.

In this context, the use of eutectic mixtures in the pharmaceutical field has a long-standing history and has found several applications both in drug delivery, whereas eutecticity improves drug solubility and permeability, and as reaction media in biocatalyzed reactions [107,108]. Recently, DESs and their derivatives have shown a great promising potential as drug delivery systems owing to their outstanding physico-chemical properties in terms of tunability, stability and low toxicology profiles [109,110]. In addition, some classes of DESs have been investigated as potential intrinsic therapeutic agents, showing a promising preliminary bioactivity *in vitro* against certain microorganisms and cancer cell lines. The applications of DESs as pharmaceutical tools have been a matter of a huge amount of research efforts over the last five years, and their improvement is currently under continuous development. The remarkable results achieved in this field have been recently extensively reviewed in the literature [111] and are summarized in Figure 3.

DESs represent a safer and biocompatible alternative to organic solvents for the solubilization of poorly water-soluble APIs, in particular for topical formulations. Promising results on the solubilization of different classes of drug (nonsteroidal anti-inflammatory drugs, antifungal, anesthetics and analgesics) have been reported using mostly ChCl-based eutectic mixtures. While the HBA portion of these DESs (ChCl) is a safe and non-expensive compound which fulfils most of the sustainability principles, the choice of the HBDs has to be carefully addressed since many HBD components might possess a significant toxicity. Several drugs have shown a dramatically increase of their solubility in DESs compared to water, raised up to 5400-fold for ibuprofen [112], 6400-fold for posaconazole in binary eutectic systems [113] and up to 53,600-fold for the antifungal drug itraconazole in a ternary ChCl:Glycolic acid:oxalic acid (1:1.6:0.4) deep eutectic mixture [111]. Furthermore, DESs could improve the chemical stability of APIs, as observed for aspirin [110] and β-lactams antibiotics [114]. Several factors affect the solubility of APIs in DESs, among them the HBD ratios which can increase or decrease the solubility depending on the nature of both the drug and the eutectic, as a consequence of the different substrate-environment interactions [112]. Even the addition of an appropriate proportion of water to DESs or NADESs may significantly change their physicochemical properties and APIs solubility. As an example, the solubility of the benzylisoquinoline alkaloid berberine in a series of NADESs is enhanced up to 12-fold compared to water when switching from binary eutectics, where berberine solubility is lower than in water, to quaternary NADESs including water as a component [115].

Another powerful approach which has been extensively investigated to enhance APIs’ solubility entails the incorporation of the active pharmaceutical species as a constituent of the deep eutectic mixture itself (API-DES or THEDES, Therapeutic Deep Eutectic Solvents) [116]. These eutectics can be designed using a variety of APIs (acting as HBAs or HBDs) and counterparts (e.g., metabolites) to fulfil specific therapeutic purposes [117]. API-DES formulations have shown remarkable results in the permeation enhancement of transdermal drug delivery systems. Several drugs such as ibuprofen [118], lidocaine [119] and itraconazole [120] incorporated with several permeation enhancers (terpenes or other drugs) in API-DES mixtures have shown both a remarkable solubility enhancement and an increased transdermal delivery in isotonic solution. API-DESs have been also exploited to improve some drug’s oral bioavailability (e.g., CoQ10) [121], the intestinal absorption of daidzein [122] and the solubility and permeability of several drugs such as paeonol [123], ibuprofen and aspirin [112,124]. Moreover, the development of dual-drug eutectic systems incorporating two different APIs in the same eutectic formulation has opened the way to new fascinating strategies for synergic multimodal therapies using drugs with enhanced solubilization and permeation properties [125,126,127].

API-DES have been also exploited to control drug delivery as monomers for polymer production. These eutectic systems represent a significant advance in the development of controlled drug delivery systems, since they are able to (a) provide the API and (b) act both as monomer and reaction media for the polymerization reaction as a single formulation [128]. As an example, lidocaine has been incorporated in acrylic or methacrylic acid containing DESs which, after polymerization, allowed the controlled release of the anesthetic drug triggered by several parameters such as pH and ionic strength [129]. In addition, polymeric eutectic systems provide a simpler and greener alternative method for the incorporation of drugs and polymers. Several drug delivery systems have been investigated in (bio)polymer-based API-DES mixtures, among them anticancer (doxorubicin [130], Paclitaxel [131]), anti-inflammatory (ibuprofen [132], dexamethasone [133]) and anesthetic (prilocaine [134], lidocaine [135]) drugs, using poly(vinyl alcohol) (PV) and poly(acrylic acid) (PA) polymers, ammonium salts, SPCL (starch and poly-ε-caprolactone polymeric blend), cellulose [136], poly(octanediol-co-citrate) elastomers and gelatine [137] to tune the APIs release profiles.

In addition to drug delivery applications, DESs have recently shown promising pharmaceutical activities as antimicrobial (antiviral, antibacterial and antifungal) [138] and anticancer [139] agents. Preliminary studies in this field suggest that eutectic mixtures themselves have the potential to be further deeply investigated for the development of novel bio-inspired therapeutic agents.

## 6. Recent Advances in the Employment of DESs Both as Non-Innocent Solvents and as Active Co-Catalysts

The pursuit for the setup of sustainable processes in organic synthesis is a topic of paramount importance both from the academic and the industrial points of view. In this context, DESs have emerged as excellent solvents for environmentally benign reactions [4], especially in comparison with ILs, which have shown significant toxicity and extremely difficult preparation and purification procedures in several cases. Over the last fifteen years, DESs have extensively been used as reaction media for a large number of organic transformations, namely alkylation, condensation and multicomponent [18], organometallic reactions [140], together with sporadic bio- and transition metal-catalyzed processes [74]. DESs have also proven their feasibility as media in different types of processes, being employed for polymerization reactions [141], delignification of biomass feedstocks [142] and for the extraction [143] and purification of organic compounds from complex matrixes [144].

**Figure 4:**
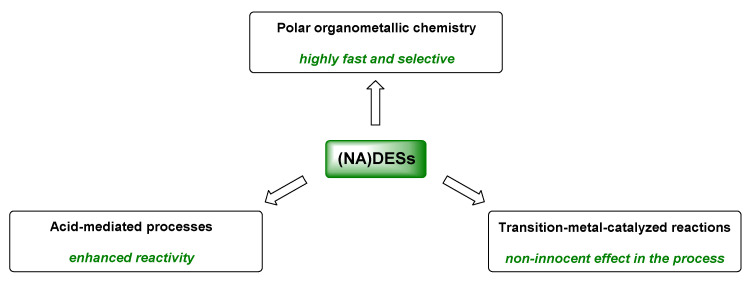
Fields of the most recent applications of DESs as solvents or co-catalysts in organic transformations.

Among the impressive number of reports on the application of DESs in organic synthesis [18], those in which at least one component of the DES strongly participates to the transformation appear as the most appealing. The active role of the DES is achieved by reacting with other molecules present in the environment or by actively promoting the process, demonstrating a non-innocent effect of the eutectic mixture on the chemistry involved. After a careful examination of the literature, the examples that have shown a peculiar or a highly different impact of DESs with respect to VOCs on the same reaction can reasonably be grouped in three main classes: (i) polar organometallic chemistry; (ii) acid-mediated and (iii) transition-metal-catalyzed processes (Figure 4). The purpose of this section is to give an overview of the most interesting and recent advances in this scenario, which is continuously evolving, highlighting the employment and the great potential of these unconventional media as true protagonists and not only as mere spectators within the aforementioned areas.

### 6.1. Polar Organometallic Chemistry

**Scheme 3:**
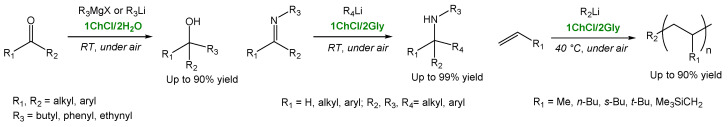
First successful organic transformations featuring organometallic reagents in DESs under air.

The unravelling of the employment of highly reactive organometallic species, i.e., Grignard and organolithium (RLi) reagents in hydrophilic protic DESs is due to the seminal work of García-Álvarez’s and Hevia’s groups. They introduced the use of organomagnesium and RLi species in DESs for the reaction with ketones [145], non-activated imines [146] and for polymerization reaction to achieve synthetically relevant polyolefins [147], reaching high yields in several cases. All the reactions proceeded with high rate at 25–40 °C under air, giving in several cases improved yields and selectivity than standard protocols performed under inert atmosphere (Scheme 3).

Hence, it was demonstrated that the rate of addition of the organometallic species could successfully compete with their protonation by the DES medium, unlocking a new reactivity for the construction of several and diverse molecular frameworks.

**Scheme 4:**
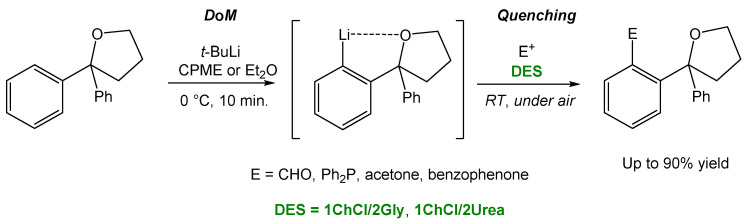
Sequential D*o*M and quenching with electrophile for selective functionalization of 2,2-diphenylTHF.

Shortly afterwards, Mallardo et al. developed a methodology for the directed *ortho*-metalation (D*o*M) of 2,2-diphenyltetrahydrofuran (THF) using *t*-BuLi in the “greener” solvent CPME and sequential quenching with several electrophiles in ChCl/Gly (1:2) at 0 °C, gaining the *o*-substitution products chemoselectively at one phenyl ring with yields up to 90% within 10 min (Scheme 4) [148].

**Scheme 5:**
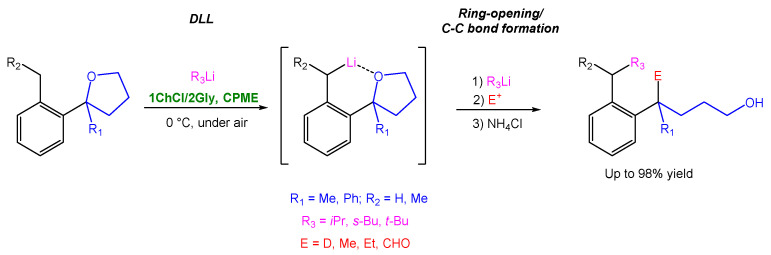
One-pot DLL/ring-opening/C–C bond formation on *o*-tolylTHFs for the synthesis of functionalized primary alcohols.

Sassone et al. demonstrated that *o*-tolylTHF derivatives could undergo unprecedented alkylative ring-opening induced by directed lateral lithiation (DLL) in CPME/DES (ChCl/Gly (1:2)) mixture [149]. After quenching with several electrophiles, a functionalized primary alcohol is obtained (Scheme 5). It is worth pointing out that in the former paper the generation of the lithiated species was described in CPME, whereas in the latter the reactions occurred in a one-pot fashion. It appears that the addition of CPME to the DES mixture is required in order to stabilize the reactive RLi species over the competitive protonolysis process under these reaction conditions (Scheme 5).

**Scheme 6:**
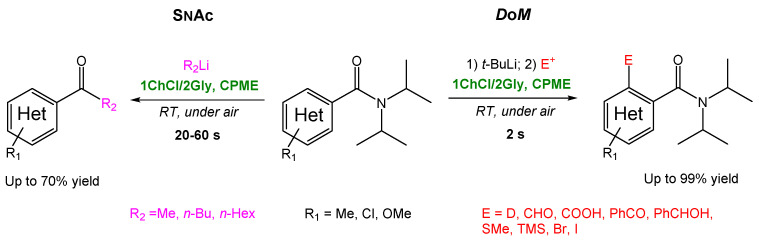
Organolithium-directed chemoselectivity in ethereal ChCl/Gly (1:2): fast D*o*M and S_N_Ac on sterically hindered benzamides in CPME/DES.

The beneficial effect of CPME in this type of processes has also been observed by Ghinato et al. [150]. The authors described the reaction between the sterically hindered (hetero)arene *N*,*N*-diisopropylamides with RLi in CPME/DES (ChCl/Gly 1:2), easily modulating the chemoselectivity by changing the nature of the organolithium reagent. When R = *t*-Bu, ultrafast D*o*M process occurred and, after addition of the proper electrophile, functionalized amides were obtained (up to 95% yield), whereas using the less hindered MeLi, *n*-BuLi and *n*-HexLi, the nucleophilic character of the lithiated species prevailed, providing the corresponding ketones (up to 70% yield) via S_N_Ac (Scheme 6). Impressively, all the reactions occurred after 2 sec for the D*o*M process and within 1 min for the S_N_Ac reaction (0–25 °C). Interestingly, the use of pure VOCs or ChCl/Gly (1:2) led to a significant decrease in conversion and chemoselectivity, suggesting that the employment of a CPME/DES mixture is mandatory to achieve high yields of the desired products.

**Scheme 7:**
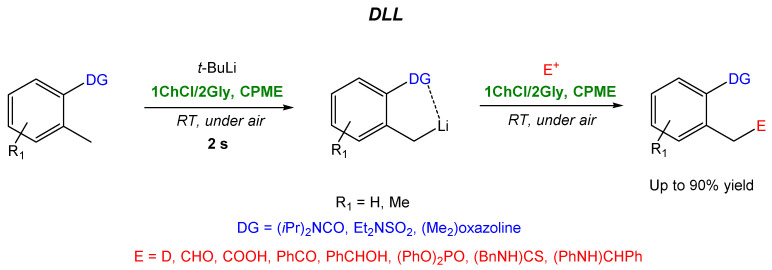
Regioselective synthesis of toluene derivatives via ultrafast DLL in ethereal ChCl/Gly (1:2).

The authors extended their methodology and investigated the lateral lithiation (LL) in DES on several *o*-tolyl-tertiary amides, sulfonamides and oxazolines, providing the DLL products functionalized with several electrophiles, over very short reaction times (Scheme 7) [151].

### 6.2. Acid-Mediated Reactions

Acid-based DESs have significantly been employed as media and reagents in different transformations, such as esterification [152], polymerization reactions [153] and for biomass valorization through the cleavage of chalcogenide moieties present in lignin feedstocks [154]. An interesting overview on the chemical and technological applications of Brønsted and Lewis acid based DESs, together with a discussion of their structural and acidity characteristics is offered by the recent survey of Qin et al. [155].

**Scheme 8:**
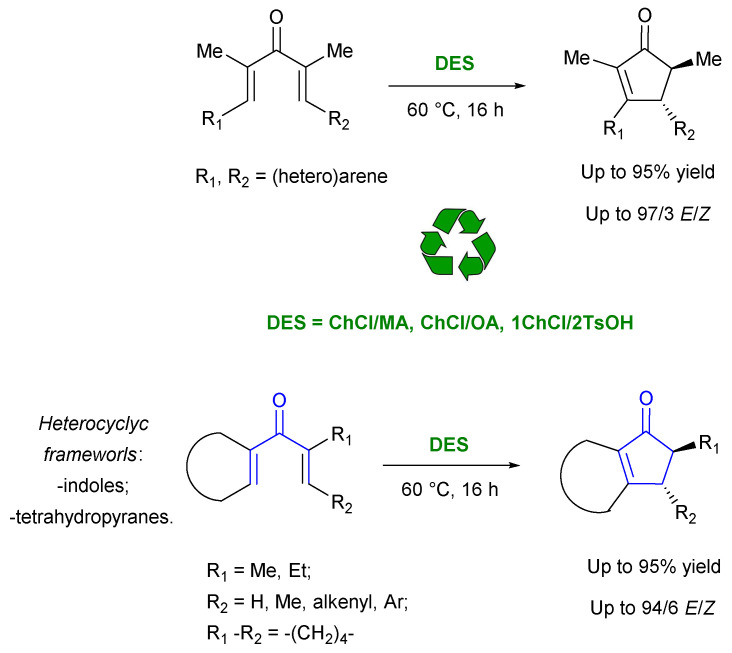
Scope of the Nazarov cyclization in acidic DESs.

Among the acid-mediated processes, the Nazarov cyclization has arisen as a facile and highly atom economic route for the obtainment of cyclopentenone derivatives from divinyl ketone precursors. The process consists in a 4π conrotatory electrocyclization promoted by Brønsted or Lewis acids. Extensive studies employing (supported) organocatalysts and transition metal complexes have been performed [156], but only one example in DESs has been reported by Nejrotti et al. [157]. From their screening of several Brønsted-acid-based DESs, the authors found out ChCl/MA (malonic acid), ChCl/OA (oxalic acid) and ChCl/TsOH (*p*-toluenesulfonic acid) to be suitable system for promoting the reaction under mild conditions and in reasonable time (60 °C, 16 h). The scope was extended to complex molecular frameworks, accessing fused (hetero)polycyclic systems present in naturally occurring compounds containing the cyclopentenone motif (Scheme 8). Interestingly, the reported data indicated that carboxylic acids such as MA are not able to mediate the Nazarov cyclization in VOCs, hinting an increasing effect on the acidity of MA when it is a part of the DES mixture. This fact could be ascribed to its peculiar structure made of a thick network of hydrogen bonds created during the formation of the eutectic system.

In addition, the reaction has proved to be performed on a gram-scale (E-factor = 19.5) and ChCl/MA has shown a good level of recyclability, mediating the process up to 4 runs with an overall acceptable yield (42%). The catalytic activity dramatically dropped at the 5th cycle, suggesting that the DES system could undergo decomposition processes throughout the reaction and the recycle conditions employed, likely decarboxylation of the acid component and trans-esterification between MA and the alcohol moiety of ChCl (*cf*. Section 1).

Very recently, the same authors reported the unprecedent Nazarov cyclization of a model divinyl ketone in two phosphonium-based DESs, (TPMPBr/EG (1:3) TPMPBr/AA (1:3)) using a two-level full factorial Design of Experiment approach for the optimization of the reaction conditions, in terms of reaction time, temperature and substrate concentration. In this case, the data indicate a strong cooperation between the two components of the DESs in promoting the Nazarov cyclization. Surface Responding Analysis (SRA) confirmed the synergic effect between the former components of the DES, which increases the expected performances of the system. Thus, conversions >90% with high chemoselectivity were reached under mild conditions (43 °C) [158].

### 6.3. Transition-Metal-Catalyzed Reactions

Lately, special attention has been devoted to the pursuit of sustainable catalytic reactions [159,160,161,162], and especially to transition-metal-mediated processes in DESs, with applications in Pd-catalyzed cross-coupling [163], Cu-catalyzed Ullmann type [164], Ru-catalyzed redox isomerization [165,166] and metathesis reactions [167]. Notwithstanding the increased sustainability in several cases, a distinct impact of the DES with respect to conventional systems (e.g., enhanced catalytic activity), is difficult to be addressed in the aforementioned examples. Particularly, two cases showing a strong participation of the DESs employed are worth mentioning and describing.

**Scheme 9:**
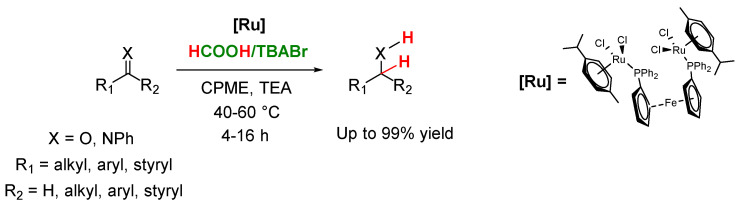
Ru-catalyzed transfer hydrogenation of carbonyl and imine compounds in TBABr/HCOOH under mild conditions.

Cavallo et al. reported for the first time the Ru-catalyzed transfer hydrogenation of carbonyl compounds and imines using the DES both as reaction medium and H_2_-source for the reduction process in the presence of TEA as the base under air [168]. After a thorough screening of different Ru-complexes and DESs, the most suitable system resulted TBABr/HCOOH, formic acid being the H_2_-source in the presence of the diphosphane complex [RuCl_2_(*p*-cymene)]_2_-*µ*-dppf as the pre-catalyst (Scheme 9). Notwithstanding the highly reactive Ru-species involved in the transfer hydrogenation processes, no significant changes in the conversion were observed when the reaction was carried out under inert atmosphere. The recovery of the hydrogenated products was achieved after 4–16 h under mild conditions (40–60 °C) and the chemoselectivity generally ranged from moderate to excellent, showing clean crude mixtures in most cases.

Interestingly, when the reduction of acetophenone was performed in VOCs (i.e., CPME, toluene, 2-MeTHF) with an equimolar mixture of HCOOH/NEt_3_, the Ru-complex was not able to catalyze the process, as well as when a solution of TBABr and HCOOH was employed, even after several hours, indicating that the presence of the peculiar DES network was crucial for attaining the catalytic reduction (Scheme 9). However, the novel system does not appear to provide an easy recycling or regeneration procedure of the DES.

**Scheme 10:**
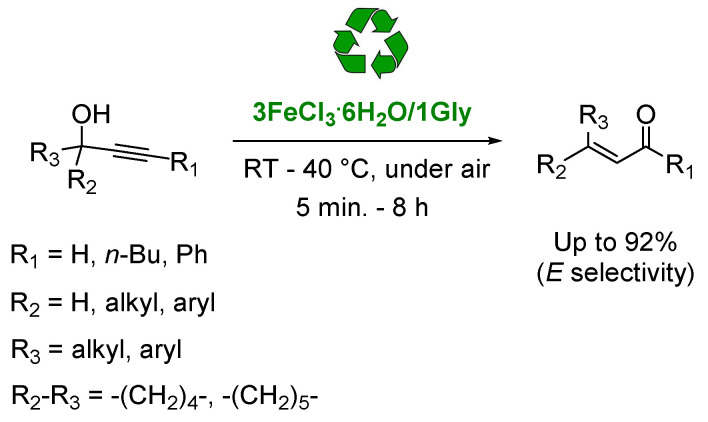
Ligand-free iron salt incorporated within the DES: Meyer-Schuster rearrangement mediated by FeCl_3_·6H_2_O/Gly (3:1).

A bright example of a green transition-metal-catalyzed transformations has recently been reported by González-Sabín’s and García-Álvarez’s groups on the Meyer-Schuster rearrangement of propargylic alcohols [169]. In this work, the cheap eutectic mixture FeCl_3_·6H_2_O/Gly (3:1) successfully mediated the reaction of non-functionalized terminal and internal alkynols to their corresponding *α*,*β*-unsaturated carbonyl compounds with full conversions and yields up to 92%. The reactions proceeded very fast (5–30 min.) in the case of 1,1-diarylalkynols at room temperature, while the rearrangement required longer reaction times to occur (1–8 h) with 1,1-dialkylalkynols at a slightly higher temperature (40 °C). FeCl_3_·6H_2_O/Gly (3:1) could be easily recycled up to ten runs without appreciable loss of catalytic activity, showing a great stability of the system employed. It is noteworthy that the incorporation of FeCl_3_·6H_2_O within the reaction medium allows the recycling of both the catalyst and the solvent (Scheme 10).

In addition, the successful although preliminary use of FeCl_3_·6H_2_O/Gly (3:1) in hydrolysis, cyclization and hydration reactions was also explored. It is worth pointing out that iron salts have scarcely been employed as catalysts for the Meyer-Schuster rearrangement in VOCs, but they required initiators or additives, higher temperatures (110–120 °C) and much longer reaction times (16 h). All the data demonstrate a reactivity enhancement of FeCl_3_·6H_2_O when part of the DES network with respect to its employment in conventional solvents. Notably, the mild reaction conditions, the facile recycle of the system and the use of a low-cost transition metal salt with no additional ligands in the place of expensive complexes confer to the process green credentials for sustainability.

## 7. Conclusions

During the last 20 years DESs have characterized a huge part of the overall research activity. The possibilities offered by the almost infinite number of eutectic combinations between HBAs and HBDs opened to the development of engineerable and modular systems. This fact, combined with the superior physical properties with respect to ILs, produced an intensive exploration of such systems in many research and applied fields, including many industrial applications. Some strategic fields of industrial interest have been herein discussed. The high levels of performance reached in ionothermal synthesis by using DESs both as solvents and as shape-directing agents are remarkable and open to the synthesis of new materials. In addition, the environmental impact of such systems when employed in VOCs treating devices is outstanding. In addition, the wide utilization of DESs in the pharmaceutical industry revealed their great promising potential as drug delivery systems to improve the pharmacokinetic properties of APIs and, in some cases, to act as APIs themselves. As a matter of fact, the exploitation of DESs is just in its early stages. In fact, as the understanding of their behavior at molecular level increases, new possibilities arise. The dual role of eutectic mixtures, as solvent and co-catalyst, together with their easy recyclability, observed in transition metal- and acid-mediated transformations could allow to upgrade many industrial processes in terms of efficiency and sustainability. Finally, the possibility to tune the structural disorder of mixtures of hydrogen bond acceptors and donors could give a relevant contribution to the development of new liquid organic semiconductors, with a cascade of industrial application currently incalculable. Regarding future outlooks, we can expect that DESs exploitation will take two main roads in the next years. From one side the relevant know-how available will be exploited for developing high effective systems in the fields of biomass conversion and in medicinal chemistry. From the other side, the extending of the eutectic concept not only to melting point but also to other physical features (as the band gap or Urbach energies) will open new application in the fields of optoelectronic, for the developing of organic liquid semiconductors of for the doping of existing systems. In addition, additional structure-activity studies are needed in order to understand how tailoring a specific hydrogen-bond network with the aim to direct the behavior of a chemical system, as an organic transformation or a catalytic process.

## Figures and Tables

**Table 1:** Classification of deep eutectic solvents.

DES Classification	General Formula	Terms
Type I	Cat^+^X^−^ · MCl_x_	M = Zn, Sn, Fe, Al, Ga
Type II	Cat^+^X^−^ · MCl_x_ · yH_2_O	M = Cr, Co, Cu, Ni, Fe
Type III	Cat^+^X^−^ · RZ	Z = CONH_2_, COOH, OH
Type IV	MCl_x_ · RZ	M = Al, Zn;Z = CONH_2_,
Type V	non ionic	COOH, OH

**Table 2:** Band gap energies of some binary DESs.

HBA ^1^	HBD ^1^	Band Gap Energy (eV)	Reference
Choline chloride (1)	Glycolic acid (1)	4.67	[44]
Choline chloride (1)	Levulinic acid (1)	5.22	[44]
Choline chloride (1)	Ethylene glycol (2)	5.92	[44]
Choline acetate (1)	Glycolic acid (1)	4.73	[44]
Choline acetate (1)	Levulinic acid (1)	4.70	[44]
Choline acetate (1)	Ethylene glycol (2)	5.30	[44]
Choline chloride (1)	Zinc chloride (2)	5.78	[44]
Choline chloride (1)	Copper chloride (2)	5.20	[44]
Choline chloride (1)	Urea (2)	5.16	[44]
Choline chloride (1)	Nickel sulphate (1)	5.18	[45]
Choline chloride (3)	Imidazole (7)	4.74	[45]
Choline chloride (2)	D-(+)-Glucose (1)	5.85	[45]
Choline chloride (1)	Glycerol (5)	5.56	[45]
Triphenylmethylphosphonium bromide (1)	Ethylene glycol (5)	5.34	[45]
Triphenylmethylphosphonium bromide (1)	Glycerol (5)	5.23	[45]

^1^ The molar equivalents of HBA and HBD are indicated in parenthesis.

**Table 3:** Fraction of esterified ChCl in mol% after heating for 2 h at different temperatures estimated through ^1^H NMR spectroscopy [61].

DES	ChCl Esterification (mol%)		
	60 °C	80 °C	100 °C
ChCl/lactic acid (1:2)	2	4	7
ChCl/levulinic acid (1:2)	2	10	17
ChCl/malic acid (1:2)	2	3	6
ChCl/oxalic acid (1:1)	0	6	17
ChCl/glutaric acid (1:1)	10	29	34
ChCl/malonic acid (1:1)	3	8	17

**Table 4:** Materials produced by ionothermal synthesis during the period 2016–2021.

Material	DES as Structure-Directing Agent
Imide-Linked Covalent Organic Frameworks	NaCl/KCl/ZnCl_2_ [65]
Layered double hydroxides	Choline chloride/urea [66]
Fe-LEV aluminophosphate molecular sieves	Succinic acid/choline chloride/tetraethylammonium bromide [67]
Ti_3_C_2_ MXene ^a^	Choline chloride and oxalic acid [68]
Triazine and heptazine polymeric carbon nitrides (PCNs)	NaCl/KCl [69]
MCM-41-supported metal catalysts	Choline chloride/glucose [70]
Fe_3_O_4_ magnetic nanoparticles	Choline chloride/urea [71]
Nanostructured ceria	Choline chloride/urea [72]
NEU20 ^b^	Choline chloride/oxalic acid [73]
Cu-doped Fe_3_O_4_ nanoparticles	Choline chloride/urea [74]
gallium phosphate Ga_3_(PO_4_)_4_(C_2_N_2_H_8_)·(H_2_C_2_N_2_H_8_)_2_·Cl	Choline chloride/imidazolinone [75]
NiCo_2_O_4_ Nanorods Decorated MoS_2_ Nanosheets	Choline chloride/urea [76]
High-silica zeolites	Tetramethylammonium chloride/1,6-hexanediol Tetrapropylammonium bromide/pentaerythritol [77] Choline chloride/urea [78]

^a^ Two-dimensional (2D) transition metal car- bides/nitrides from the 60+ group of MAX phases. ^b^ Photochromic inorganic–organic complex [C_10_N_2_H_10_]_2_[C_10_N_2_H_8_][Ga_2_(C_2_O_4_)_5_].

## Data Availability

No additional data available.
